# Enhanced cardiac substructure sparing through knowledge-based treatment planning for non-small cell lung cancer radiotherapy

**DOI:** 10.3389/fonc.2022.1055428

**Published:** 2022-12-02

**Authors:** Shadab Momin, Jonathan Wolf, Justin Roper, Yang Lei, Tian Liu, Jeffrey D. Bradley, Kristin Higgins, Xiaofeng Yang, Jiahan Zhang

**Affiliations:** Department of Radiation Oncology and Winship Cancer Institute, Emory University, Atlanta, GA, United States

**Keywords:** knowledge based treatment planning, cardiac substructures, non-small cell lung cancer radiotherapy, machine learning, cardiac toxicities

## Abstract

Radiotherapy (RT) doses to cardiac substructures from the definitive treatment of locally advanced non-small cell lung cancers (NSCLC) have been linked to post-RT cardiac toxicities. With modern treatment delivery techniques, it is possible to focus radiation doses to the planning target volume while reducing cardiac substructure doses. However, it is often challenging to design such treatment plans due to complex tradeoffs involving numerous cardiac substructures. Here, we built a cardiac-substructure-based knowledge-based planning (CS-KBP) model and retrospectively evaluated its performance against a cardiac-based KBP (C-KBP) model and manually optimized patient treatment plans. CS-KBP/C-KBP models were built with 27 previously-treated plans that preferentially spare the heart. While the C-KBP training plans were created with whole heart structures, the CS-KBP model training plans each have 15 cardiac substructures (coronary arteries, valves, great vessels, and chambers of the heart). CS-KBP training plans reflect cardiac-substructure sparing preferences. We evaluated both models on 28 additional patients. Three sets of treatment plans were compared: (1) manually optimized, (2) C-KBP model-generated, and (3) CS-KBP model-generated. Plans were normalized to receive the prescribed dose to at least 95% of the PTV. A two-tailed paired-sample *t*-test was performed for clinically relevant dose-volume metrics to evaluate the performance of the CS-KBP model against the C-KBP model and clinical plans, respectively. Overall results show significantly improved cardiac substructure sparing by CS-KBP in comparison to C-KBP and the clinical plans. For instance, the average left anterior descending artery volume receiving 15 Gy (V15 Gy) was significantly lower (p < 0.01) for CS-KBP (0.69 ± 1.57 cc) compared to the clinical plans (1.23 ± 1.76 cc) and C-KBP plans (1.05 ± 1.68 cc). In conclusion, the CS-KBP model significantly improved cardiac-substructure sparing without exceeding the tolerances of other OARs or compromising PTV coverage.

## Introduction

Radiation therapy (RT) for patients receiving definitive treatment for locally advanced non-small cell lung cancers (NSCLC) has been linked to post-RT cardiac toxicities (1). A number of promising phase II trials reported increased tumor control through radiation dose escalation *via* stereotactic fractionations (2–4). The Radiation Therapy Oncology Group (RTOG) 0617 phase III dose escalation trial compared 74 Gy with the standard 60 Gy dose with concurrent chemotherapy (5). In this study, the higher prescription dose resulted in worse overall survival. Furthermore, cardiac volumes receiving 5 Gy and 30 Gy were found to be linked with higher death rates. In a multivariate analysis, an increased radiation dose to the heart was independently linked with worse overall survival (6). More recent studies have reported the link between radiation doses to cardiac substructures and major adverse cardiac events, including mortality (1, 7). Atkins et al. reported coronary artery disease progression and the risk of ischemic events due to radiation exposure to arterial vessels, especially the left anterior descending artery (LADA) (7). Skytta et al. reported an increase of serum troponin T>30% as an endpoint to greater than 10% of LADA receiving 15 Gy (8). McWilliam et al. identified the base of the heart as a dose-sensitive region linked with poorer survival in lung cancer patients. In this study, patients receiving a mean dose of more than 8.5 Gy to this region were found to have significantly poorer survival (9). In another study, the maximum dose to the LADA of greater than 53 Gy was linked to increased risks of cardiac morbidity (10). The maximum dose to the right atrium, right coronary artery, and ascending aorta were found to be the most important factors associated with survival (11). Gore et al. reported that the pericardium mean dose and atria/ventricles volume covered by 45 Gy have detrimental effects on overall survival (12).

Clinical treatment planning is a trial-and-error process: the planner creates a treatment plan by iteratively updating optimization constraints in order to meet the target coverage while minimizing the dose to surrounding OARs. Despite best efforts from the planners, many clinical plans can be further improved in terms of OAR sparing. Such sub-optimal dose sparing can lead to increased doses to cardiac substructures and surrounding critical organs. In the past decade, knowledge-based planning (KBP) methods have been proposed to leverage knowledge embedded in prior high-quality plans to generate treatment plans for new patients (13, 14). Briefly, RapidPlan™ KBP estimates achievable DVHs for various structures included into the model, which are then translated into optimization objectives. The algorithm is divided into two parts, the model configuration and DVH estimation components. In model configuration, data extraction phase prepares the dataset for model training by extracting different geometric features of each structure. The model training phase produces a DVH estimation model for each structure. This is followed by generating estimated DVHs and optimization objectives through DVH estimation components. Previously, Harms et al. utilized RapidPlan™ KBP to improve cardiac sparing by incorporating the whole heart structure into the model training phase (15). While including the heart structure in the model would result in useful DVH predictions for the whole heart, it does not provide spatial information regarding dose distribution within the heart. We hypothesized that including cardiac substructures in the training phase of KBP would provide further dose reduction to critical cardiac substructures due to the additional spatial and geometrical features encoded into to each cardiac structure models. Therefore, the focus of this study was to build an improved KBP containing the features of each substructure (CS-KBP), in addition to other relevant OARs, to further enhance cardiac substructure sparing without compromising the target coverage. To assess the performance of a trained CS-KBP model, we used a validation dataset consisting of 28 additional patients that were not used in training the CS-KBP model.

## Materials and methods

### Patient dataset

Two separate datasets, training and validation, consisting of patients previously treated with 60 Gy in 30 fractions for NSCLC, were retrospectively collected for this study. The training cohort consisted of 27 patients who were treated with 60 Gy in 30 fractions for non-small cell lung cancer; 26 of 27 patients were included in a previous study from our institution (16), whereas the validation cohort consisted of 28 additional patients that were not included in the training phase. Patient characteristics of training and validation datasets have been listed in **Table S1**. For each patient, 15 cardiac substructures were contoured on free-breathing CT scans by resident physicians based on guidelines provided by Feng et al. (17) and reviewed by an attending physician. These substructures include the coronary arteries [LADA, left circumflex (LCFLX), left main coronary artery (LMCA), right coronary artery (RCA)], the great vessels [ascending aorta (AA), pulmonary artery (PA), superior vena cava (SVC)], the valves [atrial (AV), mitral (MV), pulmonary (PV), and tricuspid (TV)], and the chambers of the heart [left atrium (LA), right atrium (RA), right ventricle (RV), and left ventricle (LV)] The target volumes consisted of the clinical target volume (CTV) and planning target volume (PTV). These cardiac substructure contours are derived from a previous study in our group (15). However, datasets are randomly re-selected for training and validation cohorts in this study. The model constraints have also been re-tuned to improve model robustness.

### RapidPlan knowledge-based planning model

RapidPlan is a knowledge-based planning (KBP) module integrated within the Eclipse treatment planning systems (Varian medical Systems, Palo Alto, CA). RapidPlan has been widely studied and validated for various treatment sites (13, 14, 18–23). Briefly, RapidPlan utilizes anatomical and geometrical features from previously optimized plans to build a dose prediction model. The trained model is then used to predict dose volume histograms (DVHs) of various OARs and target structures for a new case of the same disease site. Detailed information on knowledge-based planning can be found in previous publications (24–26). To build a cardiac-substructure-specific KBP model (CS-KBP), we first optimized the 27 patients’ plans with their cardiac substructure preferences. To optimize plans with cardiac substructure preference, we used a line constraint for each cardiac substructure in the optimization template. Each line constraint is a collection of dose-volume point constraints placed based on RapidPlan DVH predictions.

To train the CS-KBP model, these CS-optimized plans were then used as an input along with structures including CTV, PTV, cardiac substructures, and additional organs at risk (OAR) structures (ipsilateral and contralateral lung, whole lungs, lungs cropped out of CTV, esophagus, and spinal cord). For validation, the trained CS-KBP model was then used to retrospectively generate a separate cohort of the 28 NSCLC patients’ treatment plans. In the treatment planning system (TPS), the DVH estimation tool was used to initialize the optimization to generate plans without any intervention.

Dosimetric results of this validation cohort from CS-KBP were compared against two other set of plans: clinically optimized plans and the plans generated from cardiac-based KBP model (C-KBP). Clinically optimized plans were optimized by our dosimetry team to meet desired clinical dosimetric goals for relevant OARs including the whole heart as a single structure. The C-KBP model was trained with cardiac-optimized treatment plans with the whole heart and other clinically relevant OARs as the structural input. The plan parameters including number of arcs and control points were held constant for each plan in three datasets. **Table 1** shows the summary of the three different set of plans with corresponding preferences and model inputs.

**Table 1:** Summary of three sets of validation cohort and corresponding input structures.

Training cohort (27 cases)	Validation cohort (28 cases)	Model input structures
N/A	Clinically optimized plans	N/A
Cardiac optimized treatment plans	C-KBP plans	Relevant OARs + Whole heart structure + target structures
Cardiac substructure optimized treatment plans	CS-KBP plans	Relevant OARs + 15 cardiac substructures + target structures

### Plan evaluations

The treatment plans generated by the CS-KBP model were compared against clinically optimized plans and plans generated by the C-KBP model, respectively. All dose calculations were performed in the Eclipse treatment planning software by using the Anisotropic Analytical Algorithm, version 15.6.05. To perform the dose calculation, contours were transferred through rigid registration to the average 4D CT. For dosimetric evaluations of OARs structures, clinically relevant dose volume constraints were compared across the three optimization methods. In addition to the maximum and mean doses to each cardiac substructure, we also investigated the changes in cardiac substructures’ specific dose volume metrics that have been shown to be associated with post-RT cardiac toxicities (8, 11, 12, 27, 28). We performed a paired two-tailed *t*-test to determine whether there is statistical evidence that the mean difference between paired observations is significantly different from zero. The statistical analysis was performed using Matlab’s statistical toolkit (Matlab, Mathworks Inc.). We compared the CS-KBP *vs*. C-KBP and CS-KBP *vs*. clinical plans with 95% and 99% confidence intervals for each dosimetric parameter. This test was performed with statistical significance set at p < 0.05. Finally, it is important to note that all plans were normalized to cover 100% PTV by ≥95% of the prescribed dose (57 Gy).

## Results

**Figure 1:**
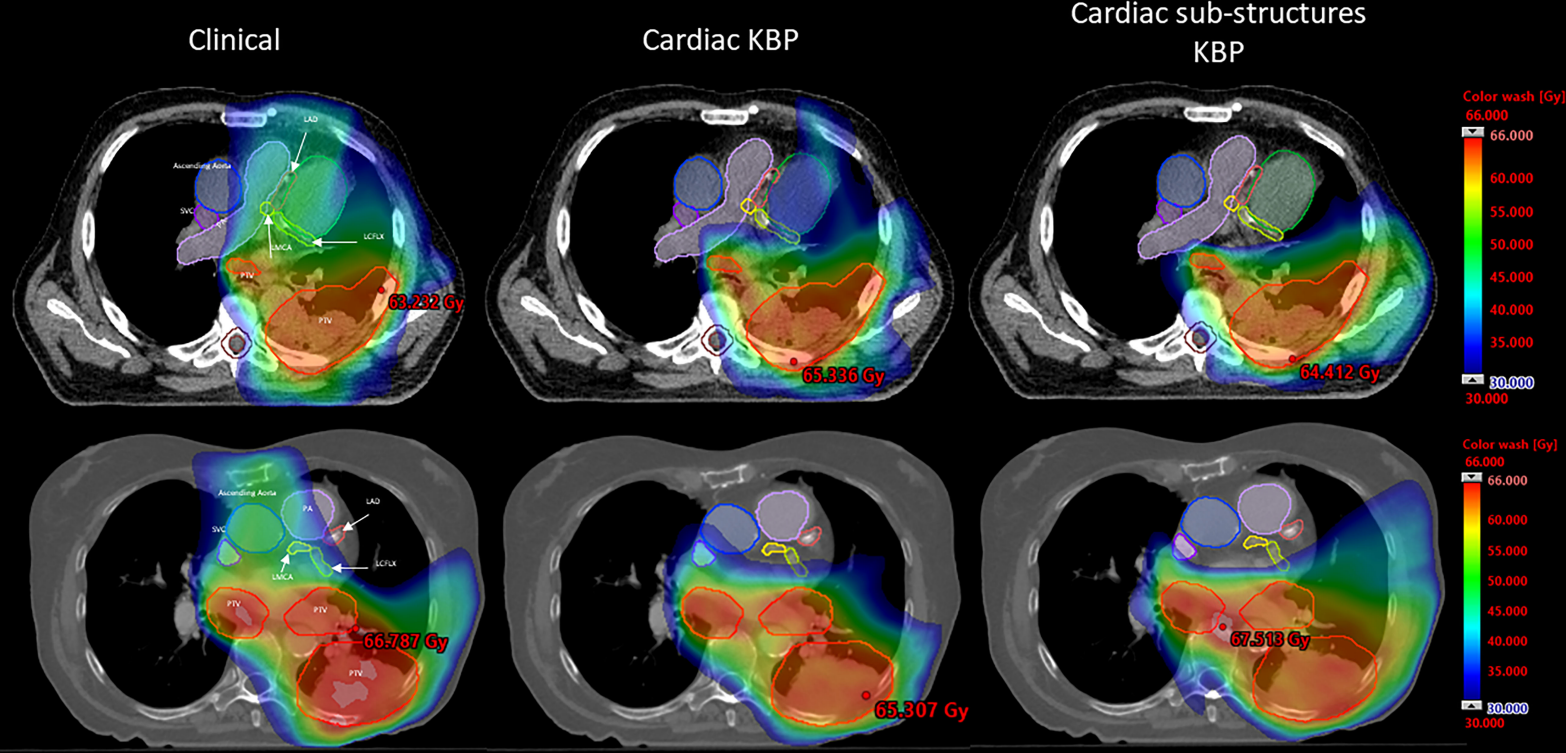
Dose distribution comparison between CS-KBP, C-KBP, and manually optimized plans for two sample cases from the validation cohort.

**Figure 2:**
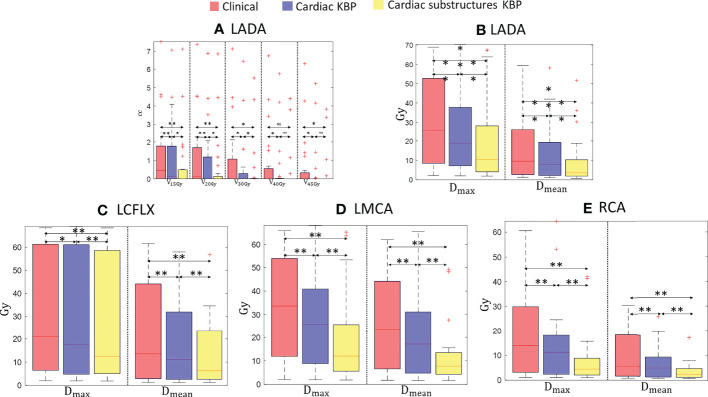
Box plots for clinically relevant dose volume metrics for LADA **(A, B)**, LCFLX **(C)**, LMCA **(D)**, and RCA **(E)** over 28 treatment plans generated by clinically optimized (orange), CKBP (purple), and CS-KBP (yellow). The central mark indicates the median, and the bottom and top edges of the box indicate 25th and 75th percentile, respectively. The whiskers extend to the most extreme data points not considered outliers, and the outliers are plotted individually using the ‘+’ marker symbol. p < 0.05 is denoted as ‘*’; p < 0.01 is denoted as ‘**’; NS = Not Significant.

**Figure 3:**
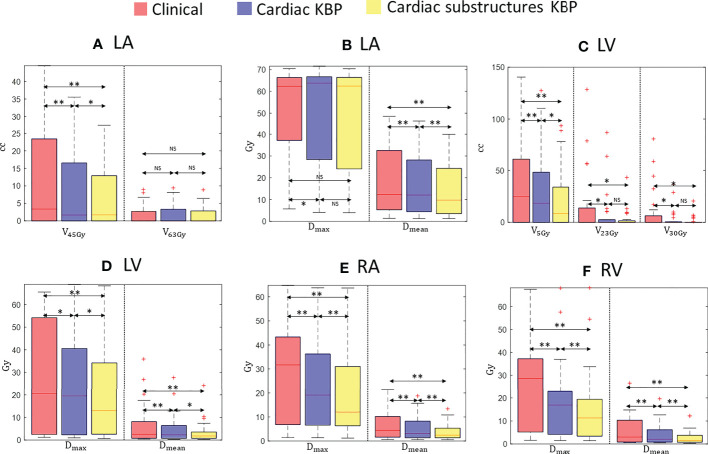
Box plots for clinically relevant dose volume metrics for LA **(A, B)**, LV **(C, D)**, RA **(E)**, and RV **(F)** over 28 treatment plans generated by clinically optimized (orange), CKBP (purple), and CS-KBP (yellow). The central mark indicates the median, and the bottom and top edges of the box indicate 25th and 75th percentile, respectively. The whiskers extend to the most extreme data points not considered outliers, and the outliers are plotted individually using the ‘+’ marker symbol. p < 0.05 is denoted as ‘*’; p < 0.01 is denoted as ‘**’; NS = Not Significant.

**Figure 4:**
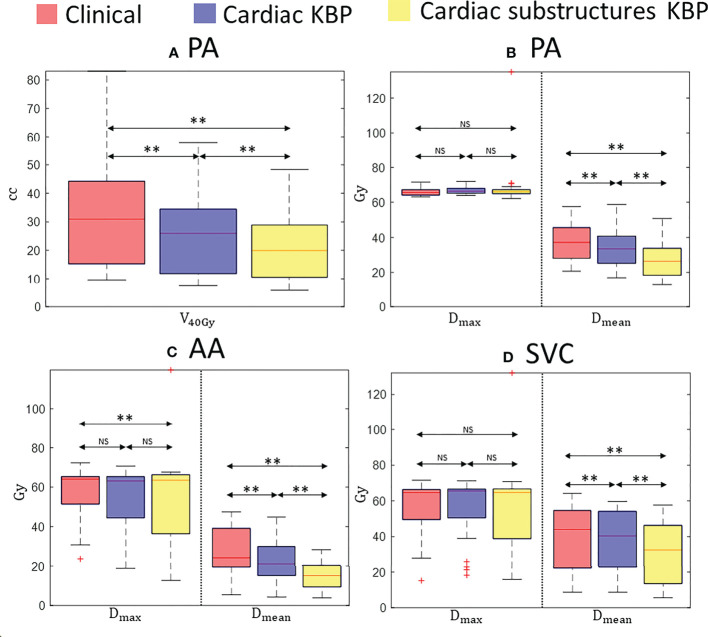
Box plots for volume of PA covered by 40 Gy isodose line ** (A) **, maximum and mean dose to PA ** (B) **, AA ** (C) **, and SVC ** (D) ** over 28 treatment plans generated by clinically optimized (orange), C-KBP (purple), and CS-KBP (yellow). The central mark indicates the median, and the bottom and top edges of the box indicate 25th and 75th percentile, respectively. The whiskers extend to the most extreme data points not considered outliers, and the outliers are plotted individually using the ‘+’ marker symbol. p < 0.05 is denoted as ‘*’; p < 0.01 is denoted as ‘**’; NS = Not Significant.

We performed qualitative (**Figure 1**) and quantitative (**Figures 2**, **3**, **4**; **S1**, **2**) comparisons among the treatment plans generated by three methods. **Figure 1** shows the dose distribution comparison among three different methods for two sample cases, each with the same CT scan slice, from the validation cohort. Clinically optimized plans are more conformal than the plans produced by C-KBP and CS-KBP for both cases, but at the expense of higher doses to coronary arteries such as LADA, LCFLX, and LMCA. While the C-KBP plan leads to better substructure sparing than clinical plan, the left circumflex coronary artery receives up to 33 Gy dose. CS-KBP plans spare cardiac substructures to a large extent with a tolerable tradeoff in plan conformity. **Table 2** shows the dosimetric comparisons among the three techniques for the two cases shown in **Figure 1**.

**Table 2:** Comparison of dosimetric parameters among three techniques for two cases shown in **Figure 1**.

Dosimetric parameters	Case 1			Case 2		
	Clinical	C-KBP	CS-KBP	Clinical	C-KBP	CS-KBP
LADA V15 Gy(cc)	2.46	1.86	1.23	4.50	4.09	0
LAD V20 Gy(cc)	1.91	0.83	0.02	4.50	3.52	0
LAD *D*_max_ (Gy)	37.77	27.68	21.32	48.36	31.96	14.43
LAD *D*_mean_ (Gy)	24.61	17.55	14.16	41.40	23.42	7.15
LCFLX *D*_max_ (Gy)	59.89	59.11	59.02	54.29	38.95	33.44
LCFLX *D*_mean_ (Gy)	45.56	28.60	26.93	47.97	33.35	21.02
LMCA *D*_max_ (Gy)	46.65	27.46	17.4	48.69	26.14	11.66
LMCA *D*_mean_ (Gy)	41.68	24.78	10.58	38.78	19.74	7.02
RCA *D*_max_ (Gy)	35.50	12.20	7.08	11.17	11.16	4.32
RCA *D*_mean_ (Gy)	19.65	7.50	3.93	5.61	9.16	2.92
AA *D*_max_ (Gy)	65.62	63.93	63.44	47.25	34.10	33.92
AA *D*_mean_ (Gy)	47.47	28.26	17.10	19.14	15.64	9.84
PA *D*_max_ (Gy)	67.53	69.25	65.73	63.55	66.1	64.23
PA *D*_mean_ (Gy)	53.02	43.66	34.56	41.99	31.18	25.07
SVC *D*_max_ (Gy)	67.05	65.52	64.09	39.81	43.30	29.56
SVC *D*_mean_ (Gy)	51.19	46.48	38.75	19.78	24.69	13.45
LA *D*_max_ (Gy)	67.99	66.61	65.67	49.86	37.97	23.35
LA *D*_mean_ (Gy)	38.54	31.24	25.58	19.73	23.54	9.04
LV *D*_max_ (Gy)	54.61	23.54	26.95	53.53	39.49	26.25
LV *D*_mean_ (Gy)	17.56	8.48	7.45	26.87	20.43	9.58
RA *D*_max_ (Gy)	35.78	20.93	11.35	17.38	18.37	9.26
RA *D*_mean_ (Gy)	10.14	7.21	4.75	7.26	10.73	4.53
RV *D*_max_ (Gy)	36.55	15.57	15.08	28.40	22.55	13.34
RV *D*_mean_ (Gy)	11.77	5.41	3.79	14.80	10.48	5.99
AV *D*_max_ (Gy)	52.96	24.97	9.73	22.58	22.38	9.39
AV *D*_mean_ (Gy)	40.15	16.49	6.54	12.73	14.99	4.77
MV *D*_max_ (Gy)	44.84	16.88	14.46	39.11	34.85	17.41
MV *D*_mean_ (Gy)	29.00	10.88	7.75	25.37	24.85	9.00
PV *D*_max_ (Gy)	49.62	24.37	7.97	27.19	16.00	8.68
PV *D*_mean_ (Gy)	38.22	21.80	5.68	18.93	10.23	3.58
TV *D*_max_ (Gy)	18.03	8.03	4.61	15.95	10.82	7.23
TV *D*_mean_ (Gy)	12.00	5.73	3.21	13.36	8.88	4.20
Lungs *D*_max_ (Gy)	67.79	66.39	67.04	63.86	65.75	64.77
Lungs *D*_mean_ (Gy)	16.14	18.40	20.43	15.08	15.81	17.04
Cord D0.03 cc (Gy)	37.79	43.76	45.39	30.36	36.61	35.56
Cord D1.2 cc (Gy)	36.11	34.87	37.46	27.42	29.12	31.43

**Figure 2A** shows that the CS-KBP model significantly reduced the volume covered by 15, 20, 30, 40, and 45 Gy isodose volumes for LADA compared to two competing methods (**Figure 2B**). The CS-KBP model also produced plans with significantly lower mean and maximum doses (9.02 Gy and 20.04 Gy) than the plans generated by the C-KBP model (12.88 Gy and 25.94 Gy) and manual optimization (16.05 Gy and 29.65 Gy). A similar trend is observed for mean doses to LCFLX (12.74 Gy *vs*. 17.68 Gy and 20.68 Gy), LMCA (11.36 Gy *vs*. 20.44 Gy and 26.74 Gy), and RCA (11.36 Gy *vs*. 20.44 Gy and 26.74 Gy) (**Figures 2C, D**). In general, CS-KBP outperformed competing methods for reducing doses to coronary arteries.

**Figure 3** outlines the doses to the chambers of the heart from the three techniques. While there was no significant differences in maximum dose and V_63 Gy_ for LA among the three techniques, CS-KBP produced plans with the significant sparing of LA measured in terms of mean dose (13.34 Gy *vs*. 16.22 Gy and 17.79 Gy) and V_45 Gy_ (6.74 cc *vs*. 7.88 cc and 11.13 cc) in comparison to treatment plans generated by the C-KBP model and manual optimizations (**Figures 3A, B**). For LV (3.55 Gy *vs*. 4.72 Gy and 6.52 Gy), RV (2.80 Gy *vs*. 4.11 Gy and 5.95 Gy) and RA (3.95 Gy *vs*. 5.59 Gy and 6.55 Gy), CS-KBP produced significant sparing across all dose volume metrics (**Figures 3C-F**).

**Figure 4** summarizes the results for the great vessels across all three methods. **Figure 4** shows a significant difference between CS-KBP and clinical plans for AA. However, **Figure 4** shows that all three methods performed comparably (p > 0.05) for maximum doses to PA and SVC. However, CS-KBP plans resulted in significantly lower mean doses to all three great vessels compared to two competing methods. As shown in **Figure S2**, CS-KBP also produced plans with significant sparing of all four cardiac valves compared to C-KBP and manually optimized plans. For whole heart structures, mean doses were 14.87 ± 5.01 Gy, 10.97 ± 3.41 Gy, and 8.95 ± 3.89 Gy for clinical, C-KBP, and CS-KBP plans, respectively. In terms of doses to the lungs, spinal cord, and esophagus, manually optimized plans largely performed better than both KBP models (**Figures S1**, **S3**). However, it is important to note that there was no violation of the clinically important dose volume constraints for these structures by treatment plans generated by the C-KBP and CS-KBP model.

## Discussion

A large number of studies in the literature have shown a correlation between post-radiotherapy cardiac toxicities and cardiac substructure irradiation (1, 3, 6, 10, 11, 29, 30). As a result, recent treatment planning efforts for thoracic cancers have been placed on sparing cardiac substructures surrounding the PTV compared to the traditional approach of sparing the heart as a single organ. Given a large number of substructures, it can be time-consuming to reach a desired treatment plan while effectively sparing substructures and other OARs. In general, it is challenging to build a KBP model for lung cancer due to variations in the location, shape, size, and orientation of PTV with respect to cardiac substructures and other critical OARs. This study developed a CS-KBP model and demonstrated its utility in the significant sparing of cardiac substructures for the VMAT of patients treated with definitive RT for locally advanced NSCLC.

For thoracic cancers, original QUANTEC recommendations are to limit the volume of the heart receiving ≥25 Gy less than 10% to keep the risk of cardiac mortality under 1% (31). Most current protocols now, however, aim to keep the mean heart dose below 20 Gy due to its correlation with a significantly higher rate of cardiac events (32). Recent works recommended substructure-specific dose volume constraints for various endpoints. For instance, *D*_max_ of 19.5 Gy to RCA has been associated with worse overall survival (11). The CS-KBP model (*D*_max_ = 8.1 ± 10.2 Gy) was able to reduce doses to RCA by 5.1 Gy and 10.1 Gy, respectively, compared to plans generated by C-KBP and manual optimization. For LADA, *D*_mean_ ≥23.8 Gy and *V*_15 Gy_ ≥10.0% has been associated with an increased percentage of serum troponin-T, which is an indicator of myocardial infarction. The CS-KBP model was able to generate plans with significantly lower mean doses to LADA compared to two competing methods (9.02 Gy *vs*. 12.88 Gy and 16.05 Gy), and also a lower volume of LADA covered with 15 Gy isodose than two competing methods (0.69 cc *vs*. 1.04 cc and 1.23 cc). In terms of great vessels, *D*_mean_ ≥8.5 Gy to AA, PA, and SVC has been linked to worse overall survival (9). While all three methods met these constraints, the CS-KBP model significantly reduced radiation doses to great vessels (**Figure 4**). However, it is important to note that great vessels of the heart were not entirely contoured within the whole heart structure for clinical and C-KBP plans, which is one limitation of this study. For LV, the volume covered by the 5 Gy (V5) isodose has been linked to overall survival and acute coronary events (12, 33). The CS-KBP model-produced plans significantly improved this constraint compared to the C-KBP and optimized plans (**Figure 3**). For LV, *D*_mean_ ≥ 6.7 Gy has been shown to increase the percentage of serum troponin (8). While all three techniques satisfied this constraint, the CS-KBP model resulted in lower *D*_mean_ to LV than C-KBP and manual optimization (3.55 Gy *vs*. 6.52 Gy and 4.72 Gy). For LV, V30 _Gy_ is also linked with symptomatic cardiac events (34). Plans generated by the CS-KBP model resulted in lower values of V30 _Gy_ compared to two competing methods (1.54 cc *vs*. 3.46 cc and 9.44 cc). In terms of atrial valve (AV), *D*_max_ ≥19.5 Gy has been associated with worse overall survival (11). While all three methods met this *D*_max_ constraint for AV, the CS-KBP model produced plans with the significantly lower *D*_max_ and *D*_mean_ to the valves of the heart (**Figure S2**). Pericardial effusion is commonly reported as one of the cardiac toxicities post thoracic radiotherapy (35). The occurrence of pericardial effusion of any grade has been reported to be higher in patients with a mean heart dose >23.45 Gy (2). While the mean heart dose constraint is met with all three techniques in this study, the proposed CS-KBP outperforms the other two techniques (Clinical: 10.68 Gy; C-KBP: 8.57 Gy; CS-KBP: 6.36 Gy).

Sparing of cardiac substructures comes with some necessary tradeoffs. Radiation doses to 65% of the lungs were significantly lower in manually optimized plans than both KBP plans. In terms of mean doses to lungs, plans generated by C-KBP (14.23 Gy) resulted in slightly lower doses than CS-KBP (14.85 Gy) and manual optimization (14.31 Gy). In terms of the dose received by 0.03 cc of the spinal cord, manually optimized plans resulted in the lowest dose (28.06 Gy) compared to C-KBP (30.77 Gy) and CS-KBP (34.26 Gy). Nonetheless, all three techniques satisfied the clinical constraints of keeping the maximum cord dose below 40 Gy. Plans generated with CS-KBP had higher plan complexity (630MU) than the ones generated *via* the C-KBP model (565MU) and manual optimization (486 MU). This tradeoff among different structures and in increased plan complexity is consistent with that of Harms et al., who found that their clinical RapidPlan™ (RP) model significantly outperformed the cardiac sparing RP model with increased plan complexity by the cardiac sparing RP model in terms of the number of monitor units (15). While CS-KBP led to the lowest overall doses to the cardiac substructures, the two competing methods also achieved the dose-volume constraints. The clinical impact of recently introduced cardiac substructure-specific dose constraints is actively being studied based on randomized trials and from real-world datasets on lung cancer (36). In contrast, there are standardized dose volume constraints for other OARs (i.e., lung and spinal cord). This study demonstrates the ability of CS-KBP to meet these standard dose volume constraints of other OARs while significantly reducing the doses to cardiac substructures. Though the direct clinical benefits from reducing the dose to cardiac substructures is yet to be validated with patient outcome data, lower doses to these cardiac substructures are expected to translate to reduced probability of radiation-induced cardiac toxicities post radiotherapy.

A limitation of the present study is that all the manually optimized plans were generated by a few dosimetrists and all thoracic OARs were contoured by a single observer. Another limitation of this study is the use of conventional CT for contouring cardiac substructures, which may introduce uncertainty due to the poor discernibility of substructures on CT images. This limitation can potentially be addressed by previous studies that leveraged the enhanced soft tissue contrast of MRI with conventional non-contrast CT to develop a deep learning-based framework (37) and a hybrid MR/CT segmentation atlas-based framework (38) for cardiac substructure segmentation. Though training the sample size was relatively small, it was within a range that had been shown to be adequate for a KBP model for thoracic radiation therapy (39). Another minor limitation of this study is a slight mismatch between the contours and the plans. Cardiac substructures were contoured on free-breathing scans for higher spatial resolution and then transferred *via* rigid registration to the average 4D CT for treatment planning. Although all patients in the cohort were treated with a free breathing technique, slight differences between the free breathing scans and the 4D average scans can be expected. Nonetheless, since all three sets of plans were generated on the same datasets, the results reflect the true differences among the three techniques. Following preliminary cardiac substructure segmentations by a physician resident, all cardiac substructures were verified by an attending physician. While we did not perform a robust evaluation for the quality of segmentation due to the nature of this study; different strategies such as percent overlap of observer volumes, a robust atlas-based method, etc. can be utilized clinically to ensure high quality segmentation of cardiac substructures.

All treatment plans were generated within one institution, lacking variations in terms of contouring and dosimetric protocols from multi-institutions. A validation cohort from multi-institutional data is necessary to further ensure the robustness of this model and also consider differences between planners and dosimetry protocols. Nonetheless, there was a large variation in the size of the PTV volumes in the validation cohort (454.05 ± 249.42 cc) of this study, demonstrating the feasibility of the proposed model for variable tumor sizes. In this study, all the structures were manually contoured, which may be time consuming. Various deep learning frameworks have been introduced to increase treatment planning efficiency (40). The impact of auto-contouring versus manual contouring on the prediction model will be investigated in the future studies. While KBP models have been built for various treatment sites, it has not been widely adopted for the purpose of cardiac substructure sparing (13). Overall results demonstrated the ability of proposed CS-KBP model to achieve significant cardiac substructure sparing compared to the traditional method of manual optimization as well as the KBP model based on cardiac structure alone (C-KBP).

## Conclusion

The proposed approach can aid future researchers to implement the knowledge-based planning module to effectively spare cardiac substructures while satisfying the clinical constraints of other OARs and target coverage and also further enhance treatment planning efficiency. This model may offer reduced planning time, improve plan quality, and should be studied in the prospective setting as a way to improve outcomes in patients with locally advanced lung cancer.

## Data availability statement

The original contributions presented in the study are included in the article/**Supplementary Material**. Further inquiries can be directed to the corresponding author.

## Author contributions

SM: First authorship. JW, JR, YL, TL, JB, KH, XY: Co-authorship. JZ: Senior authorship. All authors contributed to the article and approved the submitted version.

## Conflict of interest

The authors declare that the research was conducted in the absence of any commercial or financial relationships that could be construed as a potential conflict of interest.

## Publisher’s note

All claims expressed in this article are solely those of the authors and do not necessarily represent those of their affiliated organizations, or those of the publisher, the editors and the reviewers. Any product that may be evaluated in this article, or claim that may be made by its manufacturer, is not guaranteed or endorsed by the publisher.
