# Supplementary material for: Enhanced cardiac substructure sparing through knowledge-based treatment planning for non-small cell lung cancer radiotherapy
Source: Front Oncol. 2022 Dec 2;12:1055428. doi: 10.3389/fonc.2022.1055428 (PMC9755869; doi:10.3389/fonc.2022.1055428)
Supplement: Supplementary file 1 [file DataSheet_1.docx]

**Fig. S1.** Box plot of clinically relevant dose volume metrics for lungs, spinal cord, and esophagus over 29 treatment plans generated by clinically optimized (orange), C-KBP (purple), and CS-KBP (yellow). D_0.03cc_ and D_1.2cc_ represents dose received by 0.03cc and 1.2cc of spinal cord.

**Fig. S2.** Maximum and mean dose to all four cardiac valves from 28 treatment plans generated by clinically optimized (orange), C-KBP (purple), and CS-KBP (yellow).

**Fig. S3.** Box plot of clinically relevant dose volume metrics for lungs V_20Gy_ (%) over 29 treatment plans generated by clinically optimized (orange), C-KBP (purple), and CS-KBP (yellow).
