# Supplementary material for: Enhanced cardiac substructure sparing through knowledge-based treatment planning for non-small cell lung cancer radiotherapy
Source: Front Oncol. 2022 Dec 2;12:1055428. doi: 10.3389/fonc.2022.1055428 (PMC9755869; doi:10.3389/fonc.2022.1055428)
Supplement: Supplementary file 2 [file Table_1.docx]

**Table S1: Summary of patient characteristics for training and validation datasets**

|  | **Training** | | |  |  | **Validation** | | |
| --- | --- | --- | --- | --- | --- | --- | --- | --- |
| **No**. | **Age** | **Location** | **PTV vol. (cc)** |  | **No.** | **Age** | **Location** | **PTV vol. (cc)** |
| 1 | 83 | Right lower lobe | 136.1 |  | 29 | 87 | Left lower lobe | 528.01 |
| 2 | 66 | Left lower lobe | 430.4 |  | 30 | 66 | Right upper lobe | 204.13 |
| 3 | 76 | Right upper lobe | 534.1 |  | 31 | 70 | Left lower lobe | 503.03 |
| 4 | 77 | Right middle lobe | 297.6 |  | 32 | 50 | Right upper lobe | 397.71 |
| 5 | 79 | Right upper lobe | 590.9 |  | 33 | 66 | Left lower lobe | 109.75 |
| 6 | 37 | Right lower lobe | 627.1 |  | 34 | 76 | Left upper lobe | 346.19 |
| 7 | 70 | Left lower lobe | 890.9 |  | 35 | 81 | Left bronchus/lung | 258.83 |
| 8 | 67 | Right middle lobe | 411.8 |  | 36 | 72 | Right hilum | 190.16 |
| 9 | 64 | Right middle lobe | 430.2 |  | 37 | 68 | Right upper lobe | 532.82 |
| 10 | 78 | Right upper lobe | 215.6 |  | 38 | 62 | Left upper lobe | 247.54 |
| 11 | 74 | Left upper lobe | 338.4 |  | 39 | 77 | Right lower lobe | 465.26 |
| 12 | 80 | Left upper lobe | 460.8 |  | 40 | 73 | Right upper lobe | 220.64 |
| 13 | 79 | Left lower lobe | 385.7 |  | 41 | 63 | Right upper lobe | 268.62 |
| 14 | 84 | Left lower lobe | 1030.8 |  | 42 | 66 | Right upper lobe | 976.09 |
| 15 | 75 | Left upper lobe | 770.9 |  | 43 | 82 | Left lower lobe | 209.08 |
| 16 | 83 | Right middle lobe | 196.4 |  | 44 | 71 | Right upper lobe | 341.66 |
| 17 | 86 | Left upper lobe | 216.9 |  | 45 | 65 | Left upper lobe | 635.27 |
| 18 | 40 | Left lower lobe | 474.6 |  | 46 | 67 | Bronchus and lung | 1107.94 |
| 19 | 74 | Left upper lobe | 392.2 |  | 47 | 77 | Left lower lobe | 929.36 |
| 20 | 74 | Right upper lobe | 685.6 |  | 48 | 74 | Left upper lobe | 434.46 |
| 21 | 82 | Right lower lobe | 237.3 |  | 49 | 66 | Left upper lobe | 770.86 |
| 22 | 75 | Right middle lobe | 582.6 |  | 50 | 63 | Left upper lobe | 624.98 |
| 23 | 79 | Left upper lobe | 906.8 |  | 51 | 83 | Right upper lobe | 420.32 |
| 24 | 90 | Right upper lobe | 305.1 |  | 52 | 62 | Left upper lobe | 591.34 |
| 25 | 60 | Left upper lobe | 329.3 |  | 53 | 75 | Right upper lung | 137.6 |
| 26 | 75 | Left upper lobe | 721.2 |  | 54 | 65 | Right hilum | 400.32 |
| 27 | 73 | Right upper lobe | 81.6 |  | 55 | 73 | Right hilum | 371.53 |
|  |  |  |  |  | 56 | 69 | Left upper lobe | 489.96 |
